# A Numerical Study on the Capacity Region of a Three-Layer Wiretap Network

**DOI:** 10.3390/e25121566

**Published:** 2023-11-21

**Authors:** Jiahong Wu, Nan Liu, Wei Kang

**Affiliations:** 1 National Mobile Communications Research Laboratory, Southeast University, Nanjing 211189, China; jiahongwu@seu.edu.cn; 2 School of Information Science and Engineering, Southeast University, Nanjing 211189, China; wkang@seu.edu.cn

**Keywords:** wiretap network, secret sharing, Shannon-type inequality, common information, generator matrix

## Abstract

In this paper, we study a three-layer wiretap network including the source node in the top layer, *N* nodes in the middle layer and *L* sink nodes in the bottom layer. Each sink node recovers the message generated from the source node correctly via the middle layer nodes that it has access to. Furthermore, it is required that an eavesdropper eavesdropping a subset of the channels between the top layer and the middle layer learns absolutely nothing about the message. For each pair of decoding and eavesdropping patterns, we are interested in finding the capacity region consisting of (N+1)-tuples, with the first element being the size of the message successfully transmitted and the remaining elements being the capacity of the *N* channels from the source node to the middle layer nodes. This problem can be seen as a generalization of the secret sharing problem. We show that when the number of middle layer nodes is no larger than four, the capacity region is fully characterized as a polyhedral cone. When such a number is 5, we find the capacity regions for 74,222 decoding and eavesdropping patterns. For the remaining 274 cases, linear capacity regions are found. The proving steps are: (1) Characterizing the Shannon region, an outer bound of the capacity region; (2) Characterizing the common information region, an outer bound of the linear capacity region; (3) Finding linear schemes that achieve the Shannon region or the common information region.

## 1. Introduction

The general concept of network coding was proposed by Ahlswede et al. [1] in 2000. They investigate the single-source multicast network coding problem where the message generated by the source node is required to be sent to multiple sink nodes through a noiseless network. In addition to routing, the nodes in the network can process the received information to utilize the full capacity of the network. In 2003, Li et al. [2] demonstrated through a vector space approach that linear network coding over a finite alphabet is sufficient for an optimal multicast. Independently, Koetter and Médard [3] developed an algebraic characterization of linear network coding via a matrix approach. A deterministic polynomial time algorithm for constructing a linear network code was later presented by Jaggi et al. [4]. For more background on network coding, a useful source is [5].

Cai and Yeung [6] proposed a wiretap network which incorporates information security with network coding [7,8,9,10,11,12]. In the wiretap network, a message is sent to possibly more than one legal user and needs to be protected from eavesdroppers, who may tap a set of channels in the network. More specifically, in the wiretap network, it is required that (i) all sink nodes can obtain the message correctly and (ii) the eavesdropper, who can access any one but not more than one eavesdropping set of communication channels, obtains nothing about the message. One solution of the wiretap network is that we send both the message and the random key via a linear scheme. In this way, an eavesdropper can only observe some linear combinations of the message and the random key, which is statistically independent of the message. On the other hand, every legal user can recover the message by canceling the effect of the random key.

The performance of a wiretap network scheme can be measured by the size of the message and the size of the random key. In [6], when the eavesdropper may choose to access any subset of channels of a fixed size, tight bounds were obtained. Some general bounds under arbitrary eavesdropping sets were obtained in [13], but may not be tight in general. Focusing on a simple network topology, Cheng [14] conducted a numerical study and showed the importance of characterizing the entropic region of six linear vector spaces. When focusing on the alphabet size for the existence of secure network codes, Guang and Yeung [15] developed a graph theoretic approach to improve the existing bound. Some variants of the wiretap network include universal secure multiplex network coding [16], secure network code for adaptive and active attacks [17], secure index coding [18], multiple linear combination security network coding [19], a secure network coding for multiple unicast traffic [20] and so on.

In this paper, we focus on a three-layer wiretap network where the source node in the top layer generates the random message and *N* nodes in the middle layer relay the information sent from the source node to the sink nodes in the bottom layer. The system constraint is that each sink node can recover the message correctly via the middle layer nodes it has access to. Furthermore, the eavesdropper, who can access any one but not more than one eavesdropping set of communication channels between the source and middle layer nodes, obtains nothing about the message. Such a three-layer wiretap network was initially formulated by Cai and Yeung [6] to show that the wiretap network contains secret sharing as a special case. When the eavesdropper may choose to access any subset of channels of *a fixed size*, they had obtained the optimal scheme. But when the eavesdropping pattern is an arbitrary one, the corresponding optimal scheme is unknown. Hence, the aim of our work is to explore arbitrary decoding and eavesdropping patterns and find the corresponding optimal schemes.

The fact that the three-layer wiretap network is a generalization of the secret sharing problem [21,22] can be seen as follows. A secret sharing scheme is a method to share a secret, with the help of random key, among a set of *N* participants such that the qualified sets of participants can recover the secret, while the forbidden sets of participants can know nothing about the secret. If any subset that is not a qualified set is a forbidden set, then we have the *complete access structure* scenario. The performance of a secret sharing scheme is the (average) information ratio between the size of the share and the size of the secret given an access structure. Since the number of different access structures is finite for a fixed number of participants *N*, following a case-by-case analysis, the optimal (average) information ratio can be found when N≤4 [23] for complete access structures. In the converse part, every secret sharing scheme is treated as a discrete probability distribution, thus Shannon-type inequalities, concluded from the non-negativeness of (conditional) entropy and (conditional) mutual information of any probability distribution, are used to provide a lower bound. In terms of achievability, linear schemes, where every codeword corresponds to a distribution of *N* shares, are sufficient to achieve the converse results.

For the complete access structure, when the number of participants is five, Jackson and Martin [24] had already handled most access structures. Recently, the work was moved further by introducing a new converse for linear schemes [25]. The technique behind this discovery is called the direct use of *common information* [26]. Nevertheless, the general results in the converse are far from tight, as discussed in [27].

Guided by the existing understanding of secret sharing, we let the number of middle layer nodes *N* be less than or equal to five. Unlike secret sharing, we should consider *incomplete access structures* for the three-layer wiretap network. That is, for some subsets of channels, whether it can obtain some information about the message, is not specified, which may be the circumstance when the eavesdropper has limited eavesdropping resources. In particular, when N=5, there are a total of 74,496 different decoding and eavesdropping pattern pairs that need to be investigated.

Note that the secret sharing problem focuses on the optimal (average) information ratio, which is a scalar. To characterize the optimal (average) information ratio, one bound and one explicit scheme are needed. In the three-layer wiretap network, we consider the scenario that the channels between the top layer and the middle layer are heterogeneous, that is, the capacity of each channel may be different. For a given channel capacity vector, we are interested in the maximum amount of a message that can be securely and correctly transmitted to the sink nodes in the presence of the eavesdropper. To achieve this goal, we need to fully characterize the relationship between the size of the message and channel capacities. Such a relationship in fact formulates the capacity region, whose inner and outer bounds involve several linear schemes and inequalities.

The main contributions of this paper are the numerical results of the capacity region or linear capacity region of the three-layer wiretap network and the techniques we use to find them when the number of middle-layer nodes is no larger than five. We discuss them in detail as follows:

By exhaustive numerical experiments, we draw the conclusion that when the number of middle layer nodes is no larger than four, the capacity region is fully characterized as a polyhedral cone. However, when such a number is 5, there exist 274 decoding and eavesdropping patterns where we only find the linear capacity regions. On the other hand, the capacity regions for the other 74,222 cases are obtained.

The tools and techniques used in obtaining these results are as follows:

(1) Combine an existing bound for secret sharing or a wiretap network, which says that the size of the secret is upper bounded by the sum of the sizes of non-colluding shares, and Benson’s algorithm, which is an existing projection algorithm, to obtain the Shannon region, which is the projection of the polyhedral cone formed by Shannon-type inequalities under the system constraints and, therefore, an outer bound of the capacity region;
(2) Modify Benson’s algorithm to obtain the common information region, which adds common information for the linear achievability schemes and, therefore, is an outer bound of the linear capacity region;
(3) To obtain good linear schemes of the three-layer wiretap network, we propose the incremental kernel method (IKM), which is based on the existing Marten’s method for linear secret sharing schemes but is more memory saving and efficient. However, the essence of the IKM algorithm is still a brute-force search, which fails in two cases. Then, we propose a manual method that uses Gaussian elimination to obtain optimal linear schemes for these two cases.

## 2. System Model

### 2.1. Problem Description

**Figure 1:**
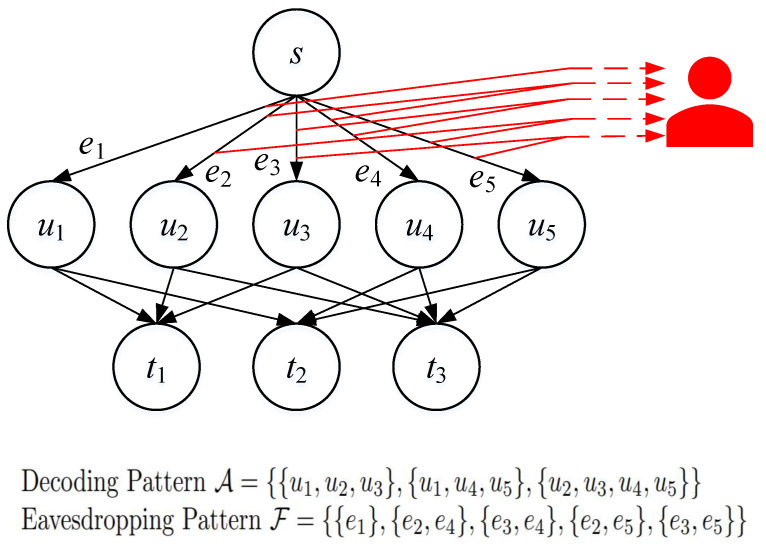
An example of the system model.

We study the model of a three-layer wiretap network, an example of which is shown in Figure 1.

Consider a directed acyclic multigraph with three layers of nodes: the top layer, the middle layer, and the bottom layer. The top layer consists of only one node, the source node, denoted as *s*. It generates a random message, *M*, which is uniformly distributed on the message set, M.

The middle layer consists of *N* nodes, denoted as $u_{1},u_{2},\cdots ,u_{N}$, and the source node connects to node $u_{n}$ by an edge $e_{n}=\left(s,u_{n}\right)$ with capacity $r_{n}$, n∈[1:N]. On Channel $e_{n}$, an index taken from an alphabet $B^{r_{n}}$ can be transmitted and is noiselessly received.

We assume that the bottom layer consists of *L* nodes, denoted as $t_{1},\cdots ,t_{L}$, and $D_{l}\subseteq \left[1:N\right]$ denotes the indices of the nodes in the middle layer to which node $t_{l}$ is connected. The channels between the middle layer nodes and the bottom layer nodes are of infinite capacity. All nodes in the bottom layer are considered sink nodes, i.e., they want to decode the message *M*, generated by the source node, without error. Let $A=\{D_{1},\cdots ,D_{L}\}$, which we call the *decoding pattern*.

There is also an eavesdropper who can access one of a collection of subsets of channels from the source node to the middle-layer nodes. More specifically, we assume that the *eavesdropping pattern* is $F=\{E_{1},\cdots ,E_{J}\}$, and the eavesdropper may access the channels between the source node and the middle layer nodes in $E_{j}$ for some j∈[1:J]. It is required that the eavesdropper knows absolutely nothing about the message *M*.

For a given $r:=(r_{1},r_{2},\ldots ,r_{N})$, we are interested in the maximum value of H(M), i.e., the maximum amount of information that can be securely and correctly transmitted to the sink nodes in the presence of the eavesdropper.

### 2.2. Arbitrary Scheme and Capacity Region

A scheme for the above three-layer wiretap network consists of a set of random local encoding mapping of the source node, $\phi_{n}\left(\cdot \right)$: $M\to B^{r_{n}}$, which maps the value of the message into an index transmitted on the channel $e_{n}$. Note that in order to securely transmit message *M* in the presence of the eavesdropper, this mapping is random. We denote $Y_{n}=\phi_{n}\left(M\right)$. We note here that encoding at the middle-layer nodes are not needed, as the output channel is of infinite capacity and, furthermore, not susceptible to eavesdropping. In other words, it is sufficient for the middle-layer node $u_{n}$ to simply forward $Y_{n}$ onto its output channels, n∈[1:N]. The scheme $\{\phi_{n}\left(\cdot \right)$: n=1,⋯,N} must satisfy the following constraints:

1. Transmission constraint: for any n∈[1:N], the entropy of $Y_{n}$ is bounded by the capacity of the channel from the source node to $u_{n}$, i.e.,
  (1) $$H\left(Y_{n}\right)\leq r_{n},\;\forall n\in \left[1:N\right].$$
2. Security constraint: for $E_{j}$ in the eavesdropping pattern F, denote $\left\{Y_{n},e_{n}\in E_{j}\right\}$ by $Y_{E_{j}}$, and given the symbols $Y_{E_{j}}$ accessed by the eavesdropper eavesdropping $E_{j}$, we have $\mathrm{Pr}\left(M=m|Y_{E_{j}}=y\right)=\mathrm{Pr}\left(M=m\right),\forall m\in M$, i.e., the eavesdropper can know absolutely nothing about the message *M*. In other words,
  (2) $$H\left(M|Y_{E_{j}}\right)=H\left(M\right),\;\forall j\in \left[1:J\right],$$
  must be satisfied.
3. Decodability constraint: for the bottom layer node $t_{l}$, who has access to $Y_{D_{l}}$, message *M* must be decoded without error, i.e.,
  (3) $$H\left(M|Y_{D_{l}}\right)=0,\;\forall l\in \left[1:L\right].$$

Since the maximum amount of information that can be correctly and securely transmitted from the source node to the destination nodes, i.e., H(M), depends on the values of the channel capacities $r_{n},n\in [1$:N], we define the *capacity region*, denoted as $C_{A,F}$, of the three-layer wiretap network as the closure of the set of any (N+1) dimension vector $\left(H\left(M\right),H\left(Y_{1}\right),\ldots ,H\left(Y_{N}\right)\right)$ corresponding to a scheme that satisfies the transmission, security and decodability constraints.

### 2.3. Linear Scheme and Linear Capacity Region

We are also interested in linear schemes for the three-layer wiretap network. In defining a linear scheme, we let the alphabet B be a finite field GF(q), where *q* is a prime power. In other words, for the edge $e_{n}$ with capacity $r_{n}$, $r_{n}$ symbols in GF(q) can be transmitted correctly over $e_{n}$.

A linear scheme $\left(r,k,V_{1},\cdots ,V_{N}\right)$ consists of the following: (1) for some fixed positive integer *r*, the message set M is taken to be GF${}^{r}\left(q\right)$, i.e.,  message *M* can be written as *r* symbols in GF(q), i.e., $M=(M_{1},\cdots ,M_{r})$; (2) for some fixed positive integer *k*, the randomness introduced by the source node to enable the secure delivery of the message to the destination nodes is denoted as *K*, which takes values in a uniform fashion in its alphabet K, which is GF${}^{k}\left(q\right)$. This means that the randomness *K* can be written as *k* symbols in GF(q) as $K=(K_{1},\cdots ,K_{k})$; (3) the source node performs linear coding, i.e., for each channel $e_{n}$, the linear coding coefficient is denoted by the matrix $V_{n}$ of size $\left(r+k\right)\times r_{n}$, where each element is in GF(q). Hence, the vector transmitted on channel $e_{n}$ is $Y_{n}=\left[\begin{array}{cccccc} M_{1} & \cdots & M_{r} & K_{1} & \cdots & K_{k} \end{array}\right]V_{n}$, which consists of $r_{n}$ elements and, therefore, does not exceed the capacity of the edge $e_{n}$. Thus, the transmission constraint, i.e., (1), is satisfied. The linear scheme must also satisfy the security constraint and the decodability constraint. Under the assumption of linear schemes, the security constraint (2) becomes

$$\begin{array}{c} \mathrm{rank}\left(\left[\begin{array}{cc} V_{M} & V_{E_{j}} \end{array}\right]\right)=\mathrm{rank}\left(V_{M}\right)+\mathrm{rank}\left(V_{E_{j}}\right),\;\forall j\in \left[1:J\right], \end{array}$$

where rank(·) denotes the rank of a matrix, $V_{M}$ is the matrix whose column vectors are associated with the message, i.e., $V_{M}=\left[\begin{array}{c} I_{r}\\ 0_{k\times r} \end{array}\right]$, and $V_{E_{j}}$ is the juxtaposition of $V_{n},n\in E_{j}$. Under the assumption of linear schemes, the decodability constraint (3) becomes

$$\begin{array}{c} \mathrm{rank}\left(\left[\begin{array}{cc} V_{M} & V_{D_{l}} \end{array}\right]\right)=\mathrm{rank}\left(V_{D_{l}}\right),\;\forall l\in \left[1:L\right], \end{array}$$

where $V_{D_{l}}$ is the juxtaposition of $V_{n},n\in D_{l}$.

We define the *linear capacity region*, denoted as $C_{A,F}^{l}$, of the three-layer wiretap network as the closure of the set of any (N+1) dimension vector $\left(r,r_{1},\ldots ,r_{N}\right)$ corresponding to a linear scheme that satisfies the transmission, security and decodability constraints.

## 3. Preliminaries

In order to characterize the capacity region (linear capacity region) for the three-layer wiretap network, we need to find its inner and outer bounds. For the capacity region, the outer bound we use is found via Shannon-type inequalities, and we call this outer bound the *Shannon region*. For the linear capacity region, the outer bound we use is found via common information, and we call this outer bound the *common information region*. The inner bound is found by explicit linear schemes. To make the paper self-contained, we first present some preliminaries on the Shannon region and the common information region.

### 3.1. The Shannon Region

The N+1 random variables of interest for any scheme for the three-layer wiretap network is $\left(M,Y_{1},\cdots ,Y_{N}\right)$. Note that for any probability distribution with N+1 discrete random variables, we can extract $2^{N+1}-1$ entropies, corresponding to $2^{N+1}-1$ different non-empty combinations of these random variables, and arrange them into a vector h. Denote $H_{N+1}$ as a $\left(2^{N+1}-1\right)$ dimension Euclidean space whose coordinates are labeled by $h_{a}$, $\emptyset \neq a\subseteq O:=\{M,Y_{1},\ldots ,Y_{N}\}$. The set of all such vectors $h\in H_{N+1}$ corresponding to a distribution is called the entropic region [28], denoted as $\Gamma^{*}$, and its closure is a convex cone [29]. In the three-layer wiretap network, the security constraint (2) and the decodability constraint (3) can be handled as homogeneous linear equations involving the coordinates from $H_{N+1}$ only. More specifically, they can be expressed as

(4) $$\begin{array}{cc} C_{1}= & \{h\in H_{N+1}:h_{M,Y_{E_{j}}}-h_{Y_{E_{j}}}-h_{M}=0,\forall j\in \left[1:J\right]\}, \end{array}$$

(5) $$\begin{array}{cc} C_{2}= & \{h\in H_{N+1}:h_{M,Y_{D_{l}}}-h_{Y_{D_{l}}}=0,\forall l\in \left[1:L\right]\}, \end{array}$$

respectively.

It is known that the closure of the entropic region is not a polyhedral cone when the number of random variables is greater than or equal to four [30]. Hence, an easy-to-calculate outer bound is considered, i.e., $\Gamma_{\left|O\right|}$. $\Gamma_{\left|O\right|}$ is a polyhedral cone represented by the intersection of two categories of closed half-spaces, named also as Shannon-type inequalities [31]:

1. Non-decreasing: If a⊆b⊆O, then $h_{a}\leq h_{b}$;
2. Submodular: $\forall a,b\subseteq O,h_{a\cup b}+h_{a\cap b}\leq h_{a}+h_{b}$.

where $h_{\emptyset }$ is taken to be 0.

Recall the definition of the capacity region, where N+1 quantities are of interest, i.e., $\left(H\left(M\right),H\left(Y_{1}\right),\ldots ,H\left(Y_{N}\right)\right)$. As for the polyhedral cone $\Gamma_{\left|O\right|}\cap C_{1}\cap C_{2}$, we likewise care about the set of N+1 coordinates, i.e., $h_{O}:=\left(h_{M},h_{Y_{1}},\ldots ,h_{Y_{N}}\right)$. To gain a more exact characterization, a suitable concept is illustrated as follows: a *projection* of a region P in $R^{n}=R^{n_{1}}\times R^{n-n_{1}}$ onto its subspace of the first $n_{1}$ coordinates is

(6) $${\mathrm{proj}}_{\left[1:n_{1}\right]}\left(P\right)=\left\{x_{1}\in R^{n_{1}}:\exists x_{2}\in R^{n-n_{1}},\left(x_{1}^{T},x_{2}^{T}\right)\in P\right\}.$$

After the above preparation, we introduce the concept of the *Shannon region*.

**Definition** **1** (Shannon Region). *Given the decoding and eavesdropping pattern pair (A,F), the Shannon region $R_{A,F}^{s}$ of this three-layer wiretap network is the projection of the polyhedral cone $\Gamma_{\left|O\right|}$ formed by Shannon-type inequalities under the security constraint $C_{1}$ and the decodability constraint $C_{2}$ onto the set of coordinates $h_{O}$, i.e.,  ${\mathrm{proj}}_{h_{O}}\left(\Gamma_{\left|O\right|}\cap C_{1}\cap C_{2}\right)$.*

Any scheme for the three-layer wiretap network will give rise to the corresponding N+1 random variables $\left(M,Y_{1},\cdots ,Y_{N}\right)$, which must satisfy Shannon-type inequalities, the security constraint $C_{1}$ and the decodability constraint $C_{2}$. Hence, the Shannon region $R_{A,F}^{s}$ is an outer bound on the capacity region $C_{A,F}$.

### 3.2. The Common Information Region

The N+1 matrices of interest for any linear scheme for the three-layer wiretap network is $\left(V_{M},V_{1},\cdots ,V_{N}\right)$. In a linear scheme, both security and decodability constraints are related to the ranks of certain matrices. To find the rules that the ranks must obey, we firstly build a framework like for the entropic region, i.e., we extract $2^{N+1}-1$ ranks corresponding to $2^{N+1}-1$ different non-empty combinations of these N+1 matrices and arrange them into a vector $h\in H_{N+1}$. Then, the set of all such vectors corresponding to N+1 matrices is bounded by the so-called *linear rank inequalities* [32].

We note here that each matrix of $\left(V_{M},V_{1},\cdots ,V_{N}\right)$ can be viewed as a subset of a finite-dimension vector space over a finite field, or a set of basis vectors (column-wise) of a vector subspace. In fact, Shannon-type inequalities constrain not only the entropies of discrete random variables but also the ranks of subsets of a vector space. The non-decreasing property holds since one subset is contained within another subset. Furthermore, the submodular property follows by the dimension formula ([33], Appendix A.2), i.e., $\forall a,b\subseteq O,\dim\left(V_{a}\right)+\dim\left(V_{b}\right)-\dim\left(V_{a\cup b}\right)=\dim\left(V_{a}\cap V_{b}\right)$, which is greater than or equal to $\dim(V_{a\cap b})$. Here, we use the convention that the vector subspace $V_{a}$ is spanned by the column vectors of the matrix $V_{a}$ and dim(·) denotes the dimension of a vector subspace.

However, when the number of matrices is greater than or equal to four, there exist other linear rank inequalities, e.g., an Ingleton inequality [34] for the four-matrix case, twenty-four new inequalities for five matrices [32], and the ongoing work for six matrices [35,36]. To the best of our understanding, all of the above new linear rank inequalities can be derived from the tool named as common information, whose definition is given below.

**Definition** **2** (Common Information). *A random variable Z conveys the common information of the random variables X and Y if H(Z|X)=H(Z|Y)=0 and H(Z)=I(X;Y). We refer to these three equations as the common information constraint.*

In other words, the random variable *Z* encapsulates the mutual information of random variables *X* and *Y*. Unfortunately, given two random variables, it is not always possible to find a third one meeting the common information constraint. Nevertheless, in the context of vector spaces (or the random variables coming from them), common information does exist. More specifically, if *X* and *Y* are subspaces of a vector space, let *Z* be the intersection of *X* and *Y*, and *Z* will have the above three properties with the entropy term replaced by the dimension term. Finally, from the definition of a linear scheme in the three-layer wiretap network, where each random variable a∈O comes from the vector subspace $V_{a}$, we may conclude that common information exists.

In order to obtain new linear rank inequalities besides Shannon-type inequalities for |O| vector subspaces, we can firstly introduce a new subspace $V_{Z}$, which is the intersection of vector subspaces $V_{X},X\subseteq O$ and $V_{Y},Y\subseteq O$. Secondly, in the Euclidean space $H_{|O|+1}$, we build an intersection of three hyperplanes as follows:

(7) $$\begin{array}{cc} C_{Z}=\{h\in H_{|O|+1}: & h_{Z,X}-h_{X}=h_{Z,Y}-h_{Y}=\\ \; & h_{Z}-h_{X}-h_{Y}+h_{X\cup Y}=0\}, \end{array}$$

which corresponds to the common information constraint. Finally, some inequalities constraining the polyhedral cone ${\mathrm{proj}}_{\left[1:2^{\left|O\right|}-1\right]}\left(\Gamma_{|O|+1}\cap C_{Z}\right)$, whose $2^{\left|O\right|}-1$ coordinates do not involve the letter *Z*, may be the desired new linear rank inequalities.

Using the above trick to obtain new linear rank inequalities and thus bound the linear capacity region of the three-layer wiretap network better, we introduce an auxiliary random variable *Z* that is the common information of random variables X⊆O and Y⊆O and the corresponding intersection of three hyperplanes $C_{Z}$. As for the polyhedral cone $\Gamma_{|O|+1}\cap C_{1,2}\cap C_{Z}$, where the hyperplanes in $C_{1,2}:=C_{1}\cap C_{2}$ are extended in the Euclidean space $H_{|O|+1}$, we care about the set of N+1 coordinates $h_{O}$, similar to the case of the Shannon region. Still using the concept of projection, it follows that the polyhedral cone ${\mathrm{proj}}_{h_{O}}\left(\Gamma_{|O|+1}\cap C_{1,2}\cap C_{Z}\right)$ is an outer bound of the linear capacity region.

In obtaining the twenty-four new linear rank inequalities for five vector subspaces, it has been shown that different choices of common information lead to different inequalities [32]. Therefore, the polyhedral cone ${\mathrm{proj}}_{h_{O}}\left(\Gamma_{|O|+1}\cap C_{1,2}\cap C_{Z}\right)$ using a single choice of common information involves only part of the complete list of new linear rank inequalities, and thus may still not be tight for the linear capacity region. To obtain a tighter outer bound, a trivial idea is to build multiple projections corresponding to different choices of common information. Finally, the common information region is defined as the intersection of these projections, which is still an outer bound for the linear capacity region.

Before giving the formal definition of the common information region, some preparation is needed. Recall that O is the set of random variables essential to the three-layer wiretap network. Let an auxiliary random variable *Z* be the common information of random variables X⊆O and Y⊆O. We require that *X* and *Y* are disjointed, i.e., X∩Y=∅. In this way, we denote the number of different choices of common information for a fixed number of random variables |O| by $n_{\left|O\right|}$. In particular, $n_{6}=301$. Then, we introduce the auxiliary random variable $Z_{i}$ as the *i*-th common information of a choice of random variables X⊆O and Y⊆O and the corresponding intersection of three hyperplanes is denoted by $C_{Z_{i}}$, $i\in [1:n_{\left|O\right|}]$. Finally, the definition of the common information region is given below.

**Definition** **3** (The Common Information Region). *Given the decoding and eavesdropping pattern pair (A,F), the common information region of this three-layer wiretap network is*

$$R_{A,F}^{c}:=\cap_{i\in [1:n_{\left|O\right|}]}{\mathrm{proj}}_{h_{O}}\left(\Gamma_{|O|+1}\cap C_{1,2}\cap C_{Z_{i}}\right),$$

*where each projection ${}_{h_{O}}\left(\Gamma_{|O|+1}\cap C_{1,2}\cap C_{Z_{i}}\right)$ is the projection of the polyhedral cone $\Gamma_{|O|+1}$ formed by Shannon-type inequalities under the security constraint $C_{1}$, the decodability constraint $C_{2}$ and the common information constraint $C_{Z_{i}}$ onto the set of coordinates $h_{O}$.*

From our numerical experiments when the number of middle layer nodes is five, i.e., |O|=6, the equivalence between the common information region $R_{A,F}^{c}$, formed by single common information only, and the linear capacity region $C_{A,F}^{l}$ holds, and this is established by finding explicit linear schemes corresponding to all extreme directions of $R_{A,F}^{c}$.

## 4. Main Result

The main result of this paper is the characterization of the capacity region or the linear capacity region when the number of nodes in the middle layer is no larger than 5. It is summarized in the following:

(1) When the number of middle layer nodes N≤4 for any decoding and eavesdropping pattern pair (A,F), the capacity region of the three-layer wiretap network is found. Furthermore, the capacity region is achievable via linear schemes.
(2) When N=5, out of a total of 74,496 different decoding and eavesdropping pattern pairs (A,F), the capacity region of 74,222 of them is found and achievable via linear schemes. For the remaining 274 (A,F) pairs, the linear capacity region is found.
(3) The detailed description of the capacity region and the corresponding achievable schemes are provided on GitHub and named SS-WN.

Note that the number of different decoding and eavesdropping pattern pairs is counted after the refinement by permutation, e.g., two pairs (A={{1,2}},F={{1}})and(A={{1,2}},F={{2}}) are treated as the same one.

**Remark** **1.** *From the converse point of view, we designed the projection algorithms to obtain the Shannon region and the common information region. From the achievability point of view, we proposed an efficient algorithm and a manual method to construct 7087 linear schemes in total. The reason why the number of linear schemes is less than the number of decoding and eavesdropping pattern pairs is because two different pairs may have the subset relationship, and thus a linear scheme for the pair with more restrictions also applies to the other pair.*

**Remark** **2.** *Out of the 274 decoding and eavesdropping pattern pairs in which we only find the linear capacity regions, 17 (A,F) pairs are complete. Similarly, in the secret sharing problem, when the number of participants is five, optimal schemes that only restricted to the linear sense are proposed for eight complete access structures [25]. Such 8 access structures are included in the 17 (A,F) pairs.*

Proving the main result consists of the following steps:

1. Characterizing the *Shannon region*.
2. Characterizing the *common information region*.
3. Finding linear schemes that achieve the Shannon region or the common information region.

The methodology of the above three steps are given in Section 5.1, Section 5.2 and Section 6, respectively. In Section 5.1, we combine the existing bounds for secret sharing or the wiretap network, i.e., Set Difference Bound [13,37], and existing projection algorithm, i.e., Benson’s algorithm [38], to obtain the Shannon region. In Section 5.2, we modify Benson’s algorithm to obtain the intersection of some polyhedral cones, which leads to the construction of the common information region. In Section 6.1, we propose the IKM algorithm to obtain the linear schemes for the three-layer wiretap network, which is more memory saving and efficient than the existing construction of secret sharing schemes [39]. Meanwhile, we design a manual method in Section 6.2 to tackle two cases that the IKM algorithm fails to solve.

## 5. Obtaining Explicit Forms of the Shannon Region and the Common Information Region

### 5.1. The Shannon Region

Recall that $\Gamma_{\left|O\right|}$ is a finitely constrained polyhedral cone since the number of Shannon-type inequalities is finite for a fixed number, i.e., |O|, of random variables. According to the Minkowski–Weyl Theorem for Cones ([40], Theorem 2.10), every finitely constrained polyhedral cone has two representations: a H-representation and a V-representation. The H-representation means that a polyhedral cone P can be represented by a system of *m* linear inequalities in *n* variables, e.g.,

(8) $$\begin{array}{c} P=\{x\in R^{n}:Ax\geq 0\}, \end{array}$$

where $A\in R^{m\times n}$, which is called an inequality matrix in this paper. Meanwhile, such a polyhedral cone can also be represented by the non-negative linear combinations of *t* extreme directions, which can be treated as special vectors on the boundary of the cone, e.g.,

(9) $$\begin{array}{c} P=\{x\in R^{n}:x=R\lambda ,\lambda \geq 0\}, \end{array}$$

where $R\in R^{n\times t}$.

Then, we denote the projection of the polyhedral cone P onto the first $n_{1}$ coordinates by Q. To tackle the projection Q of the original polyhedral cone P in the H-representation onto a small number of coordinates, one idea is to work directly in the projection space and the projection is incrementally built by successive refinement of an initial approximation $Q_{0}^{\prime }$. The difference in the relationship between the initial approximation and the true projection leads to two different projection algorithms, the Convex Hull Method [41,42,43] and Benson’s algorithm [38,44]. We give an outline of Benson’s algorithm in the following.

Benson’s algorithm starts with an initial approximation that contains the true projection. For example, we can let some inequalities constraining the true projection constrain the initial approximation. Then, Benson’s algorithm gradually adds new inequalities that constrain the true projection to the approximation. The essence of the iteration is to test whether an extreme direction of the approximation also belongs to the true projection, where the negative answer leads to an inequality that will be treated as a new inequality constraining the approximation. Meanwhile, the corresponding V-representation is updated since there are new inequalities. Again, since the dimension of the projection space is small, the conversion from H-representation to V-representation can be carried in practice [45].

Benson’s algorithm has already included the method to construct the initial approximation by linear programming (LP). In our three-layer wiretap network, we can actually use some understandings of this problem to build an initial approximation that may be closer to the true projection, thus the number of iterations carried by any of the two algorithms may be smaller. We discuss this special trick according to the converse result in the following.

From the converse point of view, we can build the initial approximation for Benson’s algorithm. When considering arbitrary wiretap sets in a general wiretap network, Cheng ([13], Corollary 1) proposed a type of inequality which works in the projection space and conveys the physical meaning that the size of the message is upper bounded by the sum of capacities of non-eavesdropped channels. We call this inequality the Set Difference Bound and illustrate it formally in the following.

**Lemma** **1** (Set Difference Bound). *Given the decoding and eavesdropping pattern pair (A,F), for any decoding set A∈A and eavesdropping set F∈F,*

(10) $$\begin{array}{c} H\left(M\right)\leq \sum_{i\in A-F}H\left(Y_{i}\right). \end{array}$$

**Remark** **3.** *Recall that the Shannon region is defined as the projection of the polyhedral cone formed by Shannon-type inequalities under the security constraint and the decodability constraint onto the set of coordinates $h_{O}$. Moreover, the proof of the Set Difference Bound is also derived from Shannon-type inequalities, the security constraint and the decodability constraint in the same Euclidean space $H_{N+1}$. Finally, it follows that the Set Difference Bound forms an outer bound of the Shannon region and can be used to initialize Benson’s algorithm.*

**Remark** **4.** *In the secret sharing problem ([37], Proposition 2.2.4), the Set Difference Bound conveys the physical meaning that the size of the secret is upper bounded by the sum of sizes of non-colluding shares. In particular, for any complete access structure, the cardinality of the difference between a decoding set A and an eavesdropping set F can be one, so the Set Difference Bound is utilized to prove that the information ratio must be greater than or equal to one.*

In our numerical experiments, we adopt the Set Difference Bound to initialize Benson’s algorithm to obtain the explicit forms of the Shannon region. When the number of nodes in the middle layer is less than or equal to five, it turns out that the initial approximation equals the true projection in 64,238 cases, which is nearly 86% of the total number of different decoding and eavesdropping pattern pairs.

The original Benson’s algorithm is designed for multi-objective linear programming (MOLP) [38]. Meanwhile, the polyhedral projection problem is equivalent to MOLP, as stated in [46]. The reason is that the projection offers the full information of the sub-system related to the objectives of MOLP. For completeness, we rewrite Benson’s algorithm for the polyhedral projection problem in Algorithm 1.

The initial approximation $Q_{0}^{\prime }$ is defined by the Set Difference Bound in the non-negative orthant and the corresponding V-representation is obtained. Since the Set Difference Bound is an outer bound of the Shannon region, we have that $Q_{0}^{\prime }$ contains the true projection Q. We note here that the conversion from H-representation to V-representation can be carried by an existing Python package called pycddlib [45], due to the small size of the corresponding inequality matrix. Furthermore, the LP in Step 2 is solved by an existing commercial solver called Gurobi [47].
**Algorithm 1** Benson’s algorithm**Input:** An initial approximation $Q_{0}^{\prime }$ and the original polyhedral cone P.**Output:** The projection Q.1:Let index i=0.2:Let the temporary set S=∅. For every extreme direction d of $Q_{i}^{\prime }$, the following LP is solved:
$$\begin{array}{cc} \min_{y}\; & y^{T}A_{\left[:,1:n_{1}\right]}d \end{array}$$
(11)$$\begin{array}{cc} s.t.\; & y^{T}A_{\left[:,n_{1}+1:n\right]}=0 \end{array}$$
(12)$$\begin{array}{cc} & y\geq 0\; \end{array}$$
(13)$$\begin{array}{c} 1^{T}y=1 \end{array}$$If the optimal value is less than 0, the vector $y^{★T}A_{\left[:,1:n_{1}\right]}$ is added to S, where $y^{★}$ is the corresponding optimal solution.3:If S=∅, the true projection $Q=Q_{i}^{\prime }$ and the algorithm terminates. Otherwise, a new polyhedral cone $Q_{i+1}^{\prime }$ is formed, whose H-representation is the union of vectors in S and the whole inequality matrix of $Q_{i}^{\prime }$. Meanwhile, the V-representation of $Q_{i+1}^{\prime }$ is calculated. Then, let i=i+1 and go back to Step 2.

Basically, Benson’s algorithm gradually contracts $Q_{0}^{\prime }$ by adding new inequalities that constrain the true projection, which are explored in Step 2. In the LP of Step 2, Algorithm 1, the non-negative variable y can be used to derive an inequality that constrains the original polyhedral P in the form of $y^{T}Ax\geq 0$. Furthermore, any feasible non-negative solution $y^{\prime }$ of the system of linear Equation (11) can be utilized to form an inequality that constrains the true projection Q. More specifically, for any vector $x_{1}\in Q$, according to the definition of projection (6), there exists a vector $x_{2}\in R^{n-n_{1}}$ such that

(14) $$\begin{array}{c} y^{\prime }A{\left(x_{1}^{T},x_{2}^{T}\right)}^{T}=y^{{}^{\prime }T}A_{\left[:,1:n_{1}\right]}x_{1}\geq 0. \end{array}$$

Therefore, when the optimal value is less than 0, the inequality $y^{{}^{\prime }T}A_{\left[:,1:n_{1}\right]}x_{1}\geq 0$ constraining the true projection can be added to make the intermediate approximation $Q_{i}^{\prime }$ strictly smaller, i.e., $Q_{i+1}^{\prime }⊊Q_{i}^{\prime }$. In a polyhedral cone, the optimal value of a linear objective function may be infinitely small, so constraint (13) helps to obtain a bounded solution.

The condition for determining the termination of Benson’s algorithm is whether the approximation equals the true projection, where the equivalence means that each extreme direction of the approximation belongs to the true projection. Still based on the definition of projection (6), an  extreme direction d of the approximation $Q_{i}^{\prime }$ is in the true projection if there exists a vector $x_{2}\in R^{n-n_{1}}$ such that

(15) $$\begin{array}{c} A_{\left[:,n_{1}+1:n\right]}x_{2}\geq -A_{\left[:,1:n_{1}\right]}d. \end{array}$$

By Gale’s Theorem [40] (Theorem 2.1), the existence of such $x_{2}$ means that for any vector $y\in R^{m}$ such that y≥0 and $y^{T}A_{\left[:,n_{1}+1:n\right]}=0$, the value $-y^{T}A_{\left[:,1:n_{1}\right]}d$ must be less than or equal to zero. Coupled with the LP in Step 2, when the optimal value is greater than or equal to zero, we can see that the tested extreme direction d belongs to the true projection and the temporary set S is not updated.

Therefore, in Step 3, if the optimal value of every LP in Step 2 is greater than or equal to zero, Benson’s algorithm terminates and outputs the true projection. Otherwise, the intermediate approximation $Q_{i+1}^{\prime }$ may still be strictly bigger than the true projection and thus further refinement is inevitable.

The main cost of Benson’s algorithm is the LP in Step 2 and the representation conversion in Step 3. In practice, we run Benson’s algorithm on a personal computer with an Intel Core i9-12900K Processor and 128 gigabytes of RAM. A total of 74,880 Shannon regions are obtained within an hour.

### 5.2. The Common Information Region

Recall that the common information region is defined as the intersection of many polyhedral cones, each of which is the projection of the corresponding original polyhedral cone. Meanwhile, in obtaining the explicit forms of the Shannon region, we have already utilized the existing Benson’s algorithm to obtain the projection of the original polyhedral cone. Thus, a straightforward procedure to obtain the explicit forms of the common information region is to run Benson’s algorithm with the initialization being the Shannon region multiple times to obtain each projection and finally combine all projections to build the intersection.

Such a procedure constructs the common information region in a parallel fashion since the multiple times of running Benson’s algorithm are independent. However, we propose an algorithm that builds the common information region by running Benson’s algorithm multiple times in a serial fashion where they are correlated. According to our numerical results, this algorithm is more efficient and is based on the following observation.

**Lemma** **2.** *Let Q be the projection of the polyhedral cone P. Benson’s algorithm takes the initial approximation $Q_{0}^{\prime }$ and the original polyhedral cone P as an input and then actually outputs the intersection of the initial approximation $Q_{0}^{\prime }$ and the true projection Q, i.e., $Q_{0}^{\prime }\cap Q$.*

**Proof.**  Recall that in Benson’s algorithm, new inequalities are gradually added to the approximation. We denote the intermediate approximation in the *i*-th iteration of Benson’s algorithm by $Q_{i}^{\prime }$, and it follows that $Q_{0}^{\prime }⊋Q_{1}^{\prime }⊋\cdots ⊋Q_{k}^{\prime }$, where *k* is the number of iterations performed until termination. The proof of $Q_{k}^{\prime }=Q_{0}^{\prime }\cap Q$ is conducted by showing that the left-hand side (LHS) is inside the right-hand side (RHS) and vice versa.

The reason why the LHS is inside the RHS is that upon the termination of Benson’s algorithm, every extreme direction of the polyhedral cone $Q_{k}^{\prime }$ is inside the true projection Q, according to the discussion of (15). Meanwhile, we know that $Q_{k}^{\prime }⊊Q_{0}^{\prime }$, as mentioned above, that is, each extreme direction of $Q_{k}^{\prime }$ also belongs to $Q_{0}^{\prime }$. Hence, we have that $Q_{k}^{\prime }\subseteq Q_{0}^{\prime }\cap Q$.

The reason why the RHS is inside the LHS is that in the whole procedure of Benson’s algorithm, only inequalities that constrain the true projection Q are added to the initial approximation $Q_{0}^{\prime }$, according to the discussion of (14). In other words, the inequality matrix of the output $Q_{k}^{\prime }$ consists of the inequalities constraining $Q_{0}^{\prime }$ and some inequalities constraining Q, then we have that $Q_{0}^{\prime }\cap Q\subseteq Q_{k}^{\prime }$.    □

**Remark** **5.** *Benson’s algorithm requires that the initial approximation $Q_{0}^{\prime }$ contains the true projection Q, i.e., $Q\subseteq Q_{0}^{\prime }$. From the above lemma we can see that since the output of Benson’s algorithm is the intersection of the initial approximation and the true projection, the equivalence between the output and the true projection holds.*

**Remark** **6.** *In fact, the initial approximation $Q_{0}^{\prime }$ can be any finitely constrained polyhedral cone, that is, $Q_{0}^{\prime }$ may not contain the true projection Q. In this case, if the rest of Benson’s algorithm remains unchanged and when it terminates, the intersection of the initial approximation $Q_{0}^{\prime }$ and the true projection Q is the output, i.e., $Q_{0}^{\prime }\cap Q$.*

Since the common information region is defined as the intersection of many polyhedral cones, each of which is the projection of the corresponding original polyhedral cone, we can still adopt Benson’s algorithm to obtain the common information region based on Lemma 2 in a serial fashion, that is, the output of the previous run of Benson’s algorithm will be used as the input for the next run of Benson’s algorithm. In the following, we use the shorthand BA to denote Benson’s algorithm. Then, the formula $Q^{\prime }=\mathrm{BA}\left(Q_{0}^{\prime },P\right)$ means that Benson’s algorithm takes the initial approximation $Q_{0}^{\prime }$ and the original polyhedral cone P as an input, then the output is assigned to $Q^{\prime }$, which equals $Q_{0}^{\prime }\cap Q$ where Q is the projection of P. We name our algorithm BA-CI, that is, Benson’s Algorithm integrated with Common Information, which is illustrated as follows (Algorithm 2):
**Algorithm 2** BA-CI**Input:** The Shannon region $R_{A,F}^{s}$ and $n_{\left|O\right|}$ original polyhedral cones $\left(P^{\left(1\right)},\ldots ,P^{\left(n_{\left|O\right|}\right)}\right)$.**Output:** The common information region $R_{A,F}^{c}$.1:Let the intermediate polyhedral cone $T^{\left(0\right)}=R_{A,F}^{s}$ and i=1.2:$T^{\left(i\right)}=\mathrm{BA}\left(T^{\left(i-1\right)},P^{\left(i\right)}\right)$.3:If $i=n_{\left|O\right|}$, we have that $R_{A,F}^{c}=T^{\left(i\right)}$ and the BA-CI algorithm terminates. Otherwise, let i=i+1 and go back to Step 2.

In the setup, when given the decoding and eavesdropping pattern pair (A,F), $n_{\left|O\right|}$, original polyhedral cones $\left(P^{\left(1\right)},\ldots ,P^{\left(n_{\left|O\right|}\right)}\right)$ are prepared, each of which is formed by Shannon-type inequalities and the intersection of hyperplanes $C_{1,2}\cap C_{Z_{i}}$ where $C_{Z_{i}}$ is determined by the *i*-th common information, i∈[1:$n_{\left|O\right|}]$.

Then, we run Benson’s algorithm in series instead of the parallel implementation. More specifically, in the *i*-th iteration of the BA-CI algorithm, the information of the existing intersection of projections $R_{A,F}^{s}\cap Q^{\left(1\right)}\cap \cdots Q^{\left(i-1\right)}$ is actually utilized to accelerate the next run of Benson’s algorithm, where $Q^{\left(j\right)}$ is the projection of $P^{\left(j\right)}$. The reason is that the initial approximation $T^{\left(i-1\right)}$ is a subset of the Shannon region which is used in the straightforward procedure, and thus more extreme directions of $T^{\left(i-1\right)}$ may already belong to the true projection $Q^{\left(i\right)}$, which may lead to fewer iterations.

In the BA-CI algorithm, we have to implement Benson’s algorithm in series to utilize the intermediate result, which seems inferior compared to the straightforward procedure. However, since LP is one of the main costs of Benson’s algorithm, one trick is to implement different LPs in Step 2 of Benson’s algorithm on different CPU threads concurrently, which also takes the full advantage of the CPU performance.

In practice, it takes us nearly 73 h to obtain the common information region for 74,496 different decoding and eavesdropping pattern pairs (A,F) when the number of middle layer nodes is five. On the other hand, the time taken by the straightforward procedure is nearly 116 h.

In the pursuit of the entropic region, Csirmaz [44] uses the notion of *Copy Lemma* [28] instead of common information and implements the straightforward procedure to obtain non-Shannon-type inequalities. In this way, different choices of copy strings can be analyzed since each projection is determined exactly. However, we focused on the final result only, i.e., the intersection of many projections, which leads to the discovery of the BA-CI algorithm.

## 6. Linear Achievable Schemes

Recall that the common information region, a polyhedral cone in the Euclidean space, is an outer bound of the linear capacity region for the three-layer wiretap network. If each extreme direction of the common information region has its corresponding linear scheme $\left(r,k,V_{1},\cdots ,V_{N}\right)$, we claim that the linear capacity region is the same as the common information region. The reason is that the definition of the V-representation of the common information region is consistent with the definition of the linear capacity region. Furthermore, when the Shannon region is identical to the linear capacity region, the capacity region is also obtained since the outer bound and the inner bound meet.

In obtaining the linear scheme for secret sharing, Marten had already proposed a method [39] that can be carried by a computer. Like secret sharing, the three-layer wiretap network also involves the security constraint and the decodability constraint. So, it turns out that Marten’s method can also be used to obtain the linear scheme for the three-layer wiretap network. Moreover, we propose the IKM algorithm which shares the same core idea of Marten’s method but is more memory saving and efficient. However, two cases remain stuck due to the large complexity that the IKM algorithm cannot handle. To tackle these two cases, we employ a manual method that is based on Gaussian elimination. In the following, we will discuss these two methods, i.e., the IKM algorithm and the manual method, in detail.

### 6.1. The IKM Algorithm

Note that a linear scheme $\left(r,k,V_{1},\cdots ,V_{N}\right)$ can also be treated as a linear code with generator matrix V, where

(16) $$\begin{array}{c} V=\left[\begin{array}{cccc} V_{M} & V_{1} & \cdots & V_{N} \end{array}\right]. \end{array}$$

That is, every codeword corresponds to a distribution of the vectors transmitted on the channels between the source node and the middle layer nodes. More specifically, a codeword

$$\begin{array}{c} \left(M_{1},\cdots ,M_{r},Y_{1,1},\cdots ,Y_{1,r_{1}},\cdots ,Y_{N,r_{N}}\right)\in \mathrm{GF}{\left(q\right)}^{r+\sum_{i=1}^{i=N}r_{i}} \end{array}$$

corresponds to a distribution of *N* vectors where the message is $\left(M_{1},\cdots ,M_{r}\right)\in \mathrm{GF}{\left(q\right)}^{r}$, the vector transmitted on channel $e_{1}$ is $\left(Y_{1,1},\cdots ,Y_{1,r_{1}}\right)\in \mathrm{GF}{\left(q\right)}^{r_{1}}$ and so on.

Thus, in a linear scheme, both security and decodability constraints are related to the ranks of submatrices of the generator matrix V. Using the generator matrix formulation, Marten’s method is based on the following observations:

(1) Recall that *r* is the size of the message and *J* is the cardinality of the eavesdropping pattern. Then, for any eavesdropping set $E_{j}$, j∈[1:J], consider *r* special codewords such that the components corresponding to $Y_{E_{j}}$ are all-zero and the components corresponding to the message are non-zero. In this way, no matter what linear combinations are adopted, the eavesdropper cannot recover the message. More specifically, we arrange these rJ codewords row-wise into a matrix G and illustrate it via an example. Assume that the eavesdropping pattern F={{1},{2},{3}} and an extreme direction $\left(r,r_{1},r_{2},r_{3}\right)=\left(2,1,1,1\right)$ is considered, we have that

(17) $$\begin{array}{c} G=\left[\begin{array}{ccccc} 1 & 0 & 0 & x_{1} & x_{2}\\ 0 & 1 & 0 & x_{3} & x_{4}\\ 1 & 0 & x_{5} & 0 & x_{6}\\ 0 & 1 & x_{7} & 0 & x_{8}\\ 1 & 0 & x_{9} & x_{10} & 0\\ 0 & 1 & x_{11} & x_{12} & 0 \end{array}\right]. \end{array}$$

In addition to the constant part, G also consists of the variable part that needs to be determined later. It follows that the matrix G has already satisfied the security constraint if we analyze the corresponding rank terms.

(2) The decodability constraint asks that each column vector of the matrix $V_{M}$ corresponding to the message is a linear combination of the column vectors from the matrix $V_{D_{l}}$ where V is the generator matrix, $D_{l}$ is the *l*-th decoding set of the decoding pattern and l∈[1:L]. The linear combination coefficients are arranged into a matrix $H\in \mathrm{GF}{\left(q\right)}^{rL\times (r+\sum_{i\in [1:N]}r_{i})}$ such that ${\mathrm{VH}}^{T}=0$. Note that in H, for any decoding set $D_{l}$, the components corresponding to $Y_{\left[1:N\right]-D_{l}}$ are all-zero and the components corresponding to the message are non-zero. In this way, any sink node in the bottom layer can recover the message successfully via the linear combination. More specifically, we illustrate the matrix H via an example. Assume that the decoding pattern A={{1,2},{2,3}} and the extreme direction is $\left(r,r_{1},r_{2},r_{3}\right)=\left(2,1,1,1\right)$, we have that

(18) $$\begin{array}{c} H=\left[\begin{array}{ccccc} 1 & 0 & y_{1} & y_{2} & 0\\ 0 & 1 & y_{3} & y_{4} & 0\\ 1 & 0 & 0 & y_{5} & y_{6}\\ 0 & 1 & 0 & y_{7} & y_{8} \end{array}\right]. \end{array}$$

In addition to the constant part, H also consists of the variable part that needs to be determined later.

(3) Finally, we build a system of bilinear equations ${\mathrm{GH}}^{T}=0$, where a feasible solution over GF(q) means that the matrix G also satisfies the decodability constraint. So, it turns out that the matrix G in its row echelon form can be treated as a generator matrix.

To discuss the above observations more rigorously, we introduce an index set $I_{0}:=\{\left(i,j\right)$:i∈[1:J],j∈[1:r]}, where *r* is the size of the message and *J* is the cardinality of the eavesdropping pattern. Furthermore, let $e^{i}$ be the *i*-th unit vector in GF${\left(q\right)}^{r}$ where the *j*-th coordinate equals 1 if j=i and 0 if j≠i. Recall that $E_{i}$ is the *i*-th eavesdropping set of the eavesdropping pattern F. Then, the security constraint leads to rJ codewords $\left(e^{j},c^{i,j}\right),\left(i,j\right)\in I_{0}$ such that

(19) $$\begin{array}{c} c_{E_{i}}^{i,j}=0,\;\forall \left(i,j\right)\in I_{0}, \end{array}$$

where $c_{E_{i}}^{i,j}$ is the juxtaposition of $c_{k}^{i,j},k\in E_{i}$. On the contrary, every component of each $c_{\left[1:N\right]-E_{i}}^{i,j}$ is a variable. Actually, there is an equivalent relationship between the security constraint for a generator matrix and the existence of these rJ codewords. For more details, see ([39], Theorem 4.2).

Similarly, let an index set $I_{1}:=\{\left(i,j\right)$:i∈[1:L],j∈[1:r]} where *L* is the cardinality of the decoding pattern. Recall that $D_{i}$ is the *i*-th decoding set of the decoding pattern A. Then, the decodability constraint leads to a special matrix H formalized by rL row vectors $\left(e^{j},c^{i,j}\right),\left(i,j\right)\in I_{1}$ such that

(20) $$\begin{array}{c} c_{\left[1:N\right]-D_{i}}^{i,j}=0,\;\forall \left(i,j\right)\in I_{1}. \end{array}$$

On the contrary, every component of each $c_{D_{i}}^{i,j}$ is a variable. Actually, there is an equivalent relationship between the decodability constraint for a generator matrix and the existence of these rL row vectors. For more details, see ([39], Theorem 4.3).

Finally, a feasible solution of the system of bilinear equations ${\mathrm{GH}}^{T}=0$ leads to a generator matrix that satisfies both the security and decodability constraints, which is summarized formally in ([39], Theorem 6.7).

Note that after finding a feasible solution, some row vectors in G may be linearly dependent due to exploiting the security constraint in this expanding form. Therefore, we can perform a row-wise Gaussian elimination to obtain a minimal set of basis row vectors of the generator matrix.

Based on Marten’s method, if we assign values to the variables of the matrix G, then G has already satisfied the security constraint. After that, ${\mathrm{GH}}^{T}=0$ can be treated as rL systems of linear equations ${\mathrm{GH}}_{\left[i,:\right]}^{T},i\in [1$:rL], which plays the role of checking whether the matrix G satisfies the decodability constraint. More specifically, if each system of linear equations has a feasible solution, the decodability constraint of G holds. Otherwise, another choice of the variables in G needs to be considered.

In a finite field GF(q), the number of choices of the variables in G is finite for a fixed prime power *q*. For example, the matrix G in (17) has 12 different variables in total, which corresponds to $q^{12}$ different choices of the variables. To prepare every choice, we can build the database row by row. That is, a list $E=\{E_{1},\ldots ,E_{rJ}\}$ is introduced such that the *i*-th element $E_{i}$ is the set of all choices of the variables in the *i*-th row vector of G. For example, the first row vector of G in (17) has two variables, then $E_{1}$ has $q^{2}$ different two-dimensional arrays where each component is chosen from GF(q). Moreover, let an index array $J=\{j_{1},\ldots ,j_{rJ}\}$ indicate the position in the database E, i.e., $j_{i}$ indicates the $j_{i}$-th array of the set $E_{i}$. Note that $j_{i}$ is not greater than $|E_{i}|$, which is the cardinality of $E_{i}$. So, it turns out that the database E and the index array J can also be used to traverse all possible choices of the variables in G, which is more memory saving compared to the tree storing all choices in [39].

Since the above preparation of the choices is row-wise, the procedure to test a choice for the decodability constraint is also carried row by row to avoid some unnecessary cases. Similar to the database E for the matrix G, a database $D^{0}=\left\{D_{1}^{0},\ldots ,D_{rL}^{0}\right\}$ for the matrix H is introduced, where each element $D_{i}^{0}$ stores all possible arrays corresponding to the variables in the *i*-th row vector of H. Basically, the initial few steps are as follows:

(1) The first row vector $G_{\left[1,:\right]}$ is fixed by the first array of the set $E_{1}$. Then, solve each linear equation $G_{\left[1,:\right]}H_{\left[i,:\right]}^{T}$ by exhausting the set $D_{i}^{0}$ of the database $D^{0}$. Finally, the corresponding rL solution sets are saved in a new list $D^{1}$;
(2) The second-row vector $G_{\left[2,:\right]}$ is fixed by the first array of the set $E_{2}$. To solve each new system of linear equations $G_{\left[1:2,:\right]}H_{\left[i,:\right]}^{T}$, we can actually solve the linear equation $G_{\left[2,:\right]}H_{\left[i,:\right]}^{T}$ based on the previous solution set $D_{i}^{1}$. Finally, the corresponding rL solution sets are saved in a new list $D^{2}$;
(3) If each set in the list $D^{2}$ is not empty, i.e., each new system of linear equations $G_{\left[1:2,:\right]}H_{\left[i,:\right]}^{T}$ is solvable, the procedure continues to the third-row vector of the matrix G;
(4) Otherwise, we assign the second array of the set $E_{2}$ to $G_{\left[2,:\right]}$ and solve the corresponding rL linear equations again. Note that any choice of G consisting of the first array of the set $E_{1}$ and the first array of the set $E_{2}$ is ignored in the procedure. In this sense, we claim that this procedure can avoid some unnecessary cases.

We name the above procedure as the incremental kernel method, or IKM for short, where the word incremental means that we tackle the system of bilinear equations ${\mathrm{GH}}^{T}=0$ incrementally and the word kernel means that we actually solve the system of linear equations. The detail of the IKM algorithm is as follows.

In the IKM algorithm, Step 7 means that the choice of the variables in the first *i* rows of G is feasible and we will move on to the next row.

If the current choice is not feasible, we need to consider the next choice as in Step 9, which leads to two circumstances depending on the database for the current row vector of G. In the first circumstance, where $j_{i}\leq \left|E_{i}\right|$, i.e., the set $E_{i}$ has not been fully explored, we continue to solve rL linear equations for the current row vector. However, in another circumstance, where $j_{i}>\left|E_{i}\right|$, i.e., the set $E_{i}$ has already been exhausted, we need to give up the *i*-th row vector temporarily. More specifically, in Step 12 we restore the index indicating the array for the *i*-th row vector to the initial position. Furthermore, in Step 13 we move to the previous row vector, for which the next choice is prepared as indicated in Step 14.

Finally, if the IKM algorithm (Algorithm 3) reaches Step 17, it means that the size of the finite field *q* needs to be larger or tighter converse results need to be found. Otherwise, there is a feasible solution for the matrix G and thus a linear scheme is constructed successfully.

**Remark** **7.** *Our proposed IKM algorithm is essentially the same as the search algorithm proposed by Marten in [39] (Section 5) in terms of the core idea, since both these algorithms traverse the choices row by row. But, in terms of data structure, these two algorithm are different. That is, the search algorithm walks in the tree storing all possible choices of the matrix G, while the IKM algorithm traverses the choices based on the database and the index array, which is more memory saving for a computer.*

**Algorithm 3** Incremental Kernel Method (IKM)**Input:** Two matrices G and H consisting of the constant part and the variable part, two corresponding databases E and $D^{0}$ and an index array J for E.**Output:** The matrix G full of constants or a warning.
1:Let each component of the index array J be 1 and *i*=1.2:
**while** 1≤i≤rJ **do**
3:   **if** $j_{i}\leq \left|E_{i}\right|$ **then**4:     Assign the $j_{i}$-th array of the set $E_{i}$ to the variables of $G_{\left[i,:\right]}$.5:     Obtain $D^{i}$ from $D^{i-1}$.6:     **if** $\forall k\in D^{i},k\neq \emptyset$ **then**7:        i=i+1.8:     **else**9:        $j_{i}=j_{i}+1$.10:     **end if**11:   **else**12:     $j_{i}=1$.13:     i=i−1.14:     $j_{i}=j_{i}+1$.15:   **end if**16:
**end while**
17:**if** i=0**then**18:   Raise a warning.19:
**else**
20:   Output the matrix G.21:
**end if**



Moreover, we propose two improvements as follows:

(1) Parallel computing can be integrated, e.g., split the database set $E_{1}$ into *m* parts and run on *m* threads of a CPU concurrently. In this way, more choices are explored per unit of time.
(2) We randomize the order of the arrays in each database set. In this way, we will obtain an average performance since we do not know which order is better beforehand.

The time complexity of the IKM algorithm depends on the number of choices of both G and H. Furthermore, the IKM algorithm is useful in finding the optimal linear achievable scheme in almost all cases of the decoding and eavesdropping pattern pair (A,F). However, the IKM algorithm is stuck for weeks for two extreme directions due to the large size of G and the nature of the brute force search of this algorithm. The first case is $A=\left\{A_{1},A_{2},A_{3}\right\},F=\left\{F_{1},F_{2},F_{3},F_{4},F_{5}\right\}$ with the extreme direction $d_{O}=\left(7,3,5,5,5,5\right)$, where $A_{1}=\left\{1,2,3\right\},A_{2}=\left\{1,4,5\right\},A_{3}=\left\{2,3,4,5\right\},$$F_{1}=\left\{1\right\},F_{2}=\left\{2,4\right\},F_{3}=\left\{3,4\right\},$$F_{4}=\left\{2,5\right\}$ and $F_{5}=\left\{3,5\right\}$. The second case is $A=\left\{A_{1},A_{2},A_{3},A_{4}\right\},F=\{F_{1},F_{2},F_{3},F_{4},$$F_{5},F_{6}\}$ with the extreme direction (5,6,6,6,2,5), where $A_{1}=\left\{1,2\right\},A_{2}=\left\{1,3\right\},$$A_{3}=\left\{2,3,4\right\},A_{4}=\left\{1,4,5\right\},F_{1}=\left\{1,4\right\},F_{2}=\left\{2,4\right\},F_{3}=\left\{3,4\right\},F_{4}=\left\{2,5\right\},F_{5}=\left\{3,5\right\}$ and $F_{6}=\left\{4,5\right\}$.

For these two cases, we resort to the manual method described below.

### 6.2. A Manual Method

Recall that a linear scheme $\left(r,k,V_{1},\cdots ,V_{N}\right)$ can be treated as a linear code with generator matrix $V:=\left[\begin{array}{cccc} V_{M} & V_{1} & \cdots & V_{N} \end{array}\right]$, whose special codewords are utilized in Marten’s method. Since Marten’s method fails in the two cases mentioned above, we turn our attention to the original generator matrix V. To build a generator matrix that satisfies the security and decodability constraints, two difficulties arise at first glance.

(1) How to choose an appropriate number of randomness, i.e., *k*;
(2) When *k* is fixed, the size of the generator matrix is also fixed, i.e., $\left(r+k\right)\times \left(r+\sum_{i\in [1:N]}r_{i}\right)$. Then, how to determine each component?

For the first difficulty, we can seek help from the converse part. Take the first case as an example, whose corresponding Shannon region is the same as the common information region. Recall that the Shannon region is the projection of the polyhedral cone $\Gamma_{\left|O\right|}$ formed by Shannon-type inequalities under security constraint $C_{1}$ and the decodability constraint $C_{2}$ onto the set of coordinates $h_{O}$. Then, in the polyhedral cone $\Gamma_{\left|O\right|}\cap C_{1}\cap C_{2}$, we extract the integral extreme direction containing the sub-vector $d_{O}$ and it turns out to be unique, denoted by d=[7,3,10,5,12,8,13,5,12,8,13,10,13,13,13,5,12,8,13,9,16,12,16,9,16,12,16,13,16,16,16,5,12,8,13,9,16,12,16,9,16,12,16,13,16,16,16,10,13,13,13,13,16,16,16,13,16,16,16,16,16,16,16], in the usual binary order of $d_{M},d_{Y_{1}},d_{M,Y_{1}},d_{Y_{2}},\ldots ,d_{O}$. So the number of randomness can be set to $d_{O}-d_{M}$, which is 9 in the first case.

In fact, finding a generator matrix, an arrangement of N+1 matrices $V_{M},V_{1},\ldots ,V_{N}$, whose $2^{N+1}-1$ rank terms correspond to the vector d is a representable *polymatroid* problem [32] (Section 5). Since the security and decodability constraints are related to the rank terms only, they are both already satisfied in the vector d. Finally, to tackle the second difficulty, we construct the generator matrix corresponding to d based on the following two ideas:

(1) The N+1 matrices are constructed one by one. That is, when constructing the *i*-th matrix, i≥2, the actual representations constructed for the i−1 matrices are utilized to fulfill the rank terms of $d_{\left[2^{i-1}:2^{i}-1\right]}$ simultaneously. More specifically, $2^{i-1}$ values calculated from d are needed, which are $d_{Y_{i-1}},d_{M,Y_{i-1}}-d_{M},d_{Y_{1},Y_{i-1}}-d_{Y_{1}},\ldots ,d_{M,Y_{1},\ldots ,Y_{i-1}}-d_{M,Y_{1},\ldots ,Y_{i-2}}$, and we use the shorthand $d_{Y_{i-1}},d_{Y_{i-1}|M},d_{Y_{i-1}|Y_{1}},\ldots ,d_{Y_{i-1}|M,Y_{1},\ldots ,Y_{i-2}}$. Note that for any $A\subseteq \{M,Y_{1},\ldots ,Y_{i-2}\}$, the value $d_{Y_{i-1}|A}$ means that the space spanned by the column vectors of the matrix corresponding to $A\cup \{Y_{i-1}\}$ has $d_{Y_{i-1}|A}$ more basis vectors than the space spanned by the column vectors of the matrix corresponding to *A*.
(2) Gaussian elimination is exhaustively used to divide the matrix under construction into the constant part and the variable part. The final variable part is handled by human experience or a computer carrying the brute force search.

In Gaussian elimination, there are three types of *elementary row operations* on a matrix that does not alter its rank: swapping two rows, multiplying a row by a nonzero number and adding a multiple of one row to another row. It is similar for *elementary column operations*. For a generator matrix V of the three-layer wiretap network, we give the following trivial observation:

**Lemma** **3.** *For any generator matrix V consisting of N+1 matrices $V_{M},V_{1},\ldots ,V_{N}$, there are two operations such that the corresponding $2^{N+1}-1$ rank terms of the changed form $V^{\prime }$ are the same as that of the original V:*

*1.*  *Elementary row operations on the whole matrix V.*
*2.*  *Elementary column operations on any matrix $V_{i},i\in \left\{M,1,\ldots ,N\right\}$.*

The proof is simple and directly follows from the fact that Gaussian elimination does not alter the rank of the matrix.

**Remark** **8.** *It is known that when using elementary row (column) operations, a matrix can always be transformed into the reduced row (column) echelon form, which is unique and consists of some fixed constants. We use these two operations in Lemma 3 to set some components of the generator matrix to be constants, which makes the later construction easier since we can rely on the existing actual representation.*

Take the first case as an example, we illustrate our construction procedure. Due to space limitation, we only show the first four matrices, which are in (21):

(21) $$\left[\begin{array}{cccc} \begin{array}{ccccccc} 1 & 0 & 0 & 0 & 0 & 0 & 0\\ 0 & 1 & 0 & 0 & 0 & 0 & 0\\ 0 & 0 & 1 & 0 & 0 & 0 & 0\\ 0 & 0 & 0 & 1 & 0 & 0 & 0\\ 0 & 0 & 0 & 0 & 1 & 0 & 0\\ 0 & 0 & 0 & 0 & 0 & 1 & 0\\ 0 & 0 & 0 & 0 & 0 & 0 & 1\\ 0 & 0 & 0 & 0 & 0 & 0 & 0\\ 0 & 0 & 0 & 0 & 0 & 0 & 0\\ 0 & 0 & 0 & 0 & 0 & 0 & 0\\ 0 & 0 & 0 & 0 & 0 & 0 & 0\\ 0 & 0 & 0 & 0 & 0 & 0 & 0\\ 0 & 0 & 0 & 0 & 0 & 0 & 0\\ 0 & 0 & 0 & 0 & 0 & 0 & 0\\ 0 & 0 & 0 & 0 & 0 & 0 & 0\\ 0 & 0 & 0 & 0 & 0 & 0 & 0 \end{array}\; & \begin{array}{ccc} 0 & 0 & 0\\ 0 & 0 & 0\\ 0 & 0 & 0\\ 0 & 0 & 0\\ 0 & 0 & 0\\ 0 & 0 & 0\\ 0 & 0 & 0\\ 1 & 0 & 0\\ 0 & 1 & 0\\ 0 & 0 & 1\\ 0 & 0 & 0\\ 0 & 0 & 0\\ 0 & 0 & 0\\ 0 & 0 & 0\\ 0 & 0 & 0\\ 0 & 0 & 0 \end{array}\; & \begin{array}{ccccc} 0 & 0 & 0 & x_{1} & x_{2}\\ 0 & 0 & 0 & x_{3} & x_{4}\\ 0 & 0 & 0 & x_{5} & x_{6}\\ 0 & 0 & 0 & x_{7} & x_{8}\\ 0 & 0 & 0 & x_{9} & x_{10}\\ 0 & 0 & 0 & x_{11} & x_{12}\\ 0 & 0 & 0 & x_{13} & x_{14}\\ 0 & 0 & 0 & y_{1} & y_{2}\\ 0 & 0 & 0 & y_{3} & y_{4}\\ 0 & 0 & 0 & y_{5} & y_{6}\\ 1 & 0 & 0 & 0 & 0\\ 0 & 1 & 0 & 0 & 0\\ 0 & 0 & 1 & 0 & 0\\ 0 & 0 & 0 & 0 & 0\\ 0 & 0 & 0 & 0 & 0\\ 0 & 0 & 0 & 0 & 0 \end{array}\; & \begin{array}{ccccc} z_{1} & z_{2} & z_{3} & z_{4} & z_{5}\\ z_{6} & z_{7} & z_{8} & z_{9} & z_{10}\\ z_{11} & z_{12} & z_{13} & z_{14} & z_{15}\\ z_{16} & z_{17} & z_{18} & z_{19} & z_{20}\\ z_{21} & z_{22} & z_{23} & z_{24} & z_{25}\\ z_{26} & z_{27} & z_{28} & z_{29} & z_{30}\\ z_{31} & z_{32} & z_{33} & z_{34} & z_{35}\\ a_{1} & a_{2} & a_{3} & b_{1} & b_{2}\\ a_{4} & a_{5} & a_{6} & b_{3} & b_{4}\\ a_{7} & a_{8} & a_{9} & b_{5} & b_{6}\\ 1 & 0 & 0 & 0 & 0\\ 0 & 1 & 0 & 0 & 0\\ 0 & 0 & 1 & 0 & 0\\ 0 & 0 & 0 & 0 & 0\\ 0 & 0 & 0 & 0 & 0\\ 0 & 0 & 0 & 0 & 0 \end{array} \end{array}\right].$$

The first matrix $V_{M}$ is always the identity matrix $I_{d_{M}}$ stacked vertically with an all-zero matrix, since for any generator matrix meeting the integral extreme direction d, it can be transformed into the reduced row echelon form by elementary row operations. We have $d_{Y_{1}|M}=3$ for the second matrix $V_{1}$, where the rank of the submatrix formed by the last nine rows must be 3 since $V_{M}$ is fixed. Then, by elementary row operations, we have an $I_{3}$ and the others are all-zero.

Since the first two matrices are fixed and $d_{Y_{2}|M,Y_{1}}=3$, in the third matrix $V_{2}$ the rank of the submatrix formed by the last six rows is 3. We use both elementary row and column operations to get an $I_{3}$, while the submatrix above $I_{3}$ and the other elements of the last six rows are all zero. As all four values for $V_{2}$ need to be filled, from $d_{Y_{2}|M}=5$, we have the rank of the last nine rows to be 5, then the *y* block is full rank and needs to be determined later. Since $d_{Y_{2}|Y_{1}}=5$, it is similar for the *x* block.

Next, consider the fourth matrix $V_{3}$, via $d_{Y_{3}|M,Y_{1},Y_{2}}=0$. We leave the last three rows to be all zero and no elementary row operation can be implemented in this matrix since the three matrices constructed before are fixed. Nevertheless, as $d_{Y_{3}|M,Y_{1}}=3$, we use elementary column operations to obtain an $I_{3}$ concatenated with an $0_{3\times 2}$ above the last three rows, and no further operation can be performed in $V_{3}$. Since $d_{Y_{3}|Y_{1},Y_{2}}=5$, we have the matrix of size 7×7, which is a concatenation of the *x* block and the *z* block, that is full rank. We can learn that the *z* block alone is full rank, as $d_{Y_{3}}=5$ and by the non-decreasing property of rank terms, it follows that $d_{Y_{3}|Y_{1}}=d_{Y_{3}|Y_{2}}=5$ is already satisfied. From $d_{Y_{3}|M}=5$, we need the *b* block to be full rank. For $d_{Y_{3}|M,Y_{2}}=1$, the matrix which is the concatenation of the *y* block, the *a* block and the *b* block needs to be full rank.

The other matrices are constructed in a similar way, i.e., leaving the final variable part to be determined by trial and error. Sometimes, these variables can be found with the assistance of a computer, which carries out a brute force search.

**Remark** **9.** *The first idea that constructs the matrices one by one is learned from [32] Section 5), where the authors faced the problem of verifying tremendous extreme directions and they handled the i-th matrix by $2^{i-1}$ values in a combinatorial style without actual numerical vector construction. On the other hand, our method uses the actual matrices constructed before for the matrix under construction. Moreover, by Gaussian elimination, we determine the constant part without loss of generality since the reduced row (column) echelon form is unique. The variable part is decided at last to satisfy all elements of the vector d simultaneously, by hand or computer with brute force search.*

**Remark** **10.** *There is a computational framework provided by [48] where group theoretic techniques for combinatorial generation are utilized. However, we were not able to get any results for weeks. In contrast, we used the manual method to tackle the two cases within four days. Still, when the number of matrices |O| is larger, we do not think this manual method is efficient due to the number of rank terms growing exponentially. Thus, we are not sure if the manual method would still work for |O|≥7, i.e., the number of middle-layer nodes is six.*

## 7. Conclusions

In this paper, we have studied the capacity region of a three-layer wiretap network that is a generalization of the secret sharing problem. By numerical experiments, we find that the capacity regions are explicit polyhedral cones when the number of middle-layer nodes is less than or equal to four. There are 274 non-tight decoding and eavesdropping pattern pairs when the number of middle-layer nodes is five, where we only obtain the linear capacity regions. The capacity regions for the other 74,222 pairs are found. In obtaining converse results, we combine an existing bound for secret sharing or the wiretap network and Benson’s algorithm to obtain the Shannon region, which is an outer bound of the capacity region. Moreover, we modify Benson’s algorithm to obtain the common information region, which is an outer bound of the linear capacity region. In achievability, we propose the IKM algorithm and a manual method to obtain the linear schemes.

## Data Availability

No new data were created or analyzed in this study. Data sharing is not applicable to this article.
